# Complete genome sequence of the molybdenum-resistant bacterium *Bacillus subtilis* strain LM 4–2

**DOI:** 10.1186/s40793-015-0118-6

**Published:** 2015-12-10

**Authors:** Xiao-Yan You, Hui Wang, Guang-Yue Ren, Jing-Jing Li, Xu Duan, Hua-Jun Zheng, Zheng-Qiang Jiang

**Affiliations:** College of Food and Bioengineering, Henan University of Science and Technology, Luoyang, P. R. China; Chinese National Human Genome Center at Shanghai, Shanghai (CHGC), 201203 P. R. China; College of Food Science and Nutritional Engineering, China Agricultural University, Beijing, P. R. China

**Keywords:** Gram-positive, Molybdate, Bioremediation, Molybdenum-resistance, *Bacillus subtilis* LM 4–2

## Abstract

*Bacillus subtilis* LM 4–2, a Gram-positive bacterium was isolated from a molybdenum mine in Luoyang city. Due to its strong resistance to molybdate and potential utilization in bioremediation of molybdate-polluted area, we describe the features of this organism, as well as its complete genome sequence and annotation. The genome was composed of a circular 4,069,266 bp chromosome with average GC content of 43.83 %, which included 4149 predicted ORFs and 116 RNA genes. Additionally, 687 transporter-coding and 116 redox protein-coding genes were identified in the strain LM 4–2 genome.

**Electronic supplementary material:**

The online version of this article (doi:10.1186/s40793-015-0118-6) contains supplementary material, which is available to authorized users.

## Introduction

*Bacillus subtilis* LM 4–2 was a molybdenum-resistant strain isolated from a molybdenum mine. It has been reported that many microbes can resist the toxicity of molybdate ion though reduction of molybdate (Mo^6+^) to Mo-blue. Molybdenum-reducing microorganisms came from a variety of genera and included the following species, *Klebsiella* spp. [1, 2], *Acidithiobacillus ferrooxidans* [3], *Enterobacter cloacae* [4], *Serratia marcescens* [5, 6], *Acinetobacter calcoaceticus* [7], *Pseudomonas* spp. [8], and *Escherichia coli* K12 [9]. The capability of molybdate-reduction presents potential possibility of molybdenum bioremediationin many polluted areas [10]. Strain LM 4–2 showed stronger resistance to molybdate (up to 850 mM Na_2_MoO_4_) than many other reported molybdenum-resistant bacteria [11, 12]. However, no information related to the molecular mechanism of molybdenum-resistance has been identified, also in genus *Bacillus*. Thus, strain LM 4–2 might be a perfect subject for us to unveil the mechanism and evaluate its possibility utilization in bioremediation. Here we present the complete genome sequence and detailed genomic features of *B. subtilis* LM 4–2.

## Organism information

### Classification and features

**Fig. 1:**
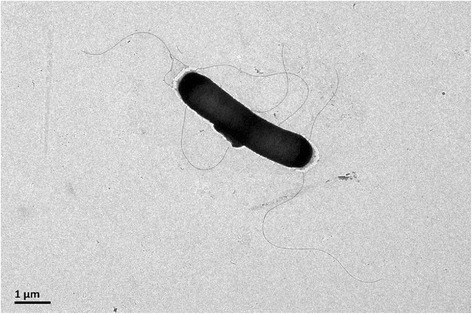
Transmission electron microscopy of strain LM 4–2. Scale bar corresponds to 1.0 μm

**Fig. 2:**
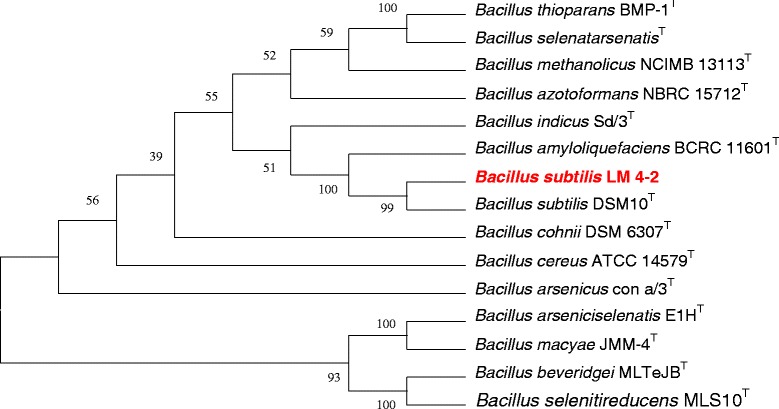
Neighbor-Joining Phylogenetic tree was built with MEGA 5 based on 16S rRNAsequences [41]. The strains and their corresponding GenBank accession numbers for 16S rDNA sequences are: **a** *Bacillus thioparans* BMP-1 (DQ371431); **b** *Bacillus selenatarsenatis* (AB262082); **c** *Bacillus methanolicus* NCIMB 13113 (AB112727); **d** *Bacillus azotoformans* NBRC 15712 (AB363732); **e** *Bacillus indicus* Sd/3 (AJ583158); **f** *Bacillus amyloliquefaciens* BCRC 11601 (NR_116022); **g** *Bacillus subtilis* 168 (NC_000964); **h** *Bacillus subtilis* PPL-SC9 (KM226924); **i** *Bacillus cohnii* DSM 6307 (X76437); **j** *Bacillus cereus* ATCC 14579 (NR_074540); **k** *Bacillus arsenicus* con a/3 (AJ606700); **l** *Bacillus arseniciselenatis* E1H (AF064705); **m** *Bacillus macyae* JMM-4 (AY032601); **n** *Bacillus beveridgei* MLTeJB (FJ825145); **o** *Bacillus selenitireducens* MLS10 (CP001791)

*Bacillus subtilis* LM 4–2 (CGMCC 1.15213) is a Gram-positive, spore-forming, rod-shaped *Bacillus* (0.3-0.5 μm wide and 3.0–4.0 μm long) with an optimum pH 6.0 and optimum temperature of 30 °C (Table 1, Fig. 1). Colonies are milky white and matte with a wrinkled surface when growth on R2A agar medium. Strictly aerobic and catalase formed. Carbon substrates utilized for growth by strain LM 4–2 included D-glucose, maltose, lactose and sucrose. Strain LM 4–2 is closely related to *Bacillus subtilis* species based on the BLAST results of 16S rRNA gene [27]. The identity of 16S rRNA gene sequence between strain LM 4–2 and type strain *B. subtilis*DSM 10^T^ is 100 %. A phylogenetic tree was constructed using the neighbor-Joining method under the default settings for complete sequence of 16S rRNA gene derived from genome of strain LM 4–2, along with the sequences of representative members of genus *Bacillus* [28–34]. The phylogenetic tree was assessed by boot-strapped for 1000 times, which is shown in Fig. 2. Average nucleotide identity (ANI), average amino acid identity (AAI) and *in silico* Genome-to-Genome Hybridization value (GGDH) were calculated between the genomes of strain LM 4–2 and other 30 *B. subtilis* species that have been completed sequenced [35–40]. Results show that strain LM 4–2 shares high ANI (>95 %, 23 of total 30), AAI (>95 %, 23 of total 30) and GGDH value (>70 %, 24 of total 30) with most of the complete sequenced *B. subtilis* species, and highest ANI (99.00 %), AAI (99.13 %) and GGDH value (92.20 % ± 1.85) with *B. subtilis* strain TO-A JPC (Additional file 1: Table S1).

**Table 1:** Classification and general features of *Bacillus subtilis* LM 4–2 according to the MIGS recommendations [13]

MIGS ID	Property	Term	Evidence code^a^
	Classification	Domain *Bacteria*	TAS [14]
		Phylum *Firmicutes*	TAS [15–17]
		Class *Bacilli*	TAS [18, 19]
		Order *Bacillales*	TAS [20, 21]
		Family *Bacillaceae*	TAS [20, 22]
		Genus *Bacillus*	TAS [20, 23, 24]
		Species *Bacillus subtilis*	TAS [25]
	Gram stain	Positive	IDA
	Cell shape	Rod-shaped	IDA
	Motility	Motile	IDA
	Sporulation	Spore-forming	NAS
	Temperature range	4–45 °C	IDA
	Optimum temperature	30 °C	IDA
	pH range; Optimum	4–9; 6.0	IDA
	Carbon source	organic carbon source	IDA
MIGS-6	Habitat	soil	IDA
MIGS-6.3	Salinity	salt tolerant	NAS
MIGS-22	Oxygen requirement	aerobic	IDA
MIGS-15	Biotic relationship	free-living	NAS
MIGS-14	Pathogenicity	non-pathogen	NAS
MIGS-4	Geographic location	Luoyang/Henan/China	IDA
MIGS-5	Sample collection	2012	IDA
MIGS-4.1	Latitude	33°55′3.21″N	
MIGS-4.2	Longitude	111°31′0.42″E	
MIGS-4.4	Altitude	1164.78	

Evidence codes - *IDA* Inferred from Direct Assay, *TAS* Traceable Author Statement (i.e., a direct report exists in the literature), *NAS* Non-traceable Author Statement (i.e., not directly observed for the living, isolated sample, but based on a generally accepted property for the species, or anecdotal evidence). These evidence codes are from the Gene Ontology project [26]

## Genome sequencing information

### Genome project history

*Bacillus subtilis* LM 4–2 was selected for sequencing due to its strong resistance to molybdate and potential utilization in bioremediation of molybdate-polluted areas. The genome sequence was deposited in GenBank under accession number CP011101 and the genome project was deposited in the Genomes on Line Database [42] under Gp0112736. Genome sequencing and annotation were performed by Chinese National Human Genome Center at Shanghai. A summary of the project was given in Table 2.

**Table 2:** Genome sequencing project information

MIGS ID	Property	Term
MIGS 31	Finishing quality	Complete
MIGS-28	Libraries used	Two libraries, 20 Kb PacBio library, 2 × 150 bpllumina library
MIGS 29	Sequencing platforms	PacBio RS II, Illumina Hi-Seq
MIGS 31.2	Fold coverage	213-and 409-fold
MIGS 30	Assemblers	HGAP, bowtie2
MIGS 32	Gene calling method	Glimmer 3.02 and GeneMark
	Locus Tag	BsLM
	Genbank ID	CP011101
	GenBank Date of Release	April 23, 2015
	GOLD ID	Gp0112736
	BIOPROJECT	PRJNA277611
MIGS 13	Source Material Identifier	CGMCC 1.15213
	Project relevance	Environmental, Bioremediation

### Growth conditions and genomic DNA preparation

*Bacillus subtilis* LM 4–2 was inoculated in 200 mL R2A medium and cultivated for 8 h at 30 °C in a shaker with speed of 200 rpm. 1.2 g of harvested cells was suspended in 5 mL TE (pH8.0) with 10 mg/mL lysozymeat 30 °C for 4 h. After centrifugation (12,000 rpm) for 10 min, genomic DNA was extracted by phenol-chloroform methods as described previously [43]. DNA was dissolved in 2 mL sterilized deionized water with a final concentration of 12.67 μg/μL and 2.04 of OD260/OD280 ratio determined by NanoDrop 2000 spectrophotometer (Thermo Scientific, USA). The genomic DNA was stored in −20 °C freezer.

### Genome sequencing and assembly

The genome of *Bacillus subtilis* LM 4–2 was sequenced by a dual sequencing approach that using a combination of PacBio RS II and Genome Analyzer IIx sequence platforms. Approximately 121,583 PacBio and 1637 million Illumina reads were generated from PacBio platform and the Illumina platform (2 × 150 bp paired-end sequencing) with average sequence coverage of 213-and 409-fold.Sequence reads from the PacBio RS II were assembled by using hierarchical genome-assembly process assembler and finally only one self-cycled supper contig was generated. The Illumina reads were quality trimmed with the CLC Genomics Workbench and then utilized for error correction of the PacBio reads by using bowtie2 (version 2.1.0) software [44].

### Genome annotation

The Glimmer 3.02 and GeneMark programs were used to predict the positions of open reading frames [45, 46]. Protein function was predicted by the following methods: 1) homology searches in the GenBank and UniProt protein database [47]; 2) function assignment searches in CDD database [48]; and 3) domain or motif searches in the Pfam databases [49]. The KEGG database was used to reconstruct metabolic pathways [50]. Ribosomal RNAs and Transfer RNAs were predicted by using RNAmmer and tRNAscan-SE programs [51, 52]. Transporters were predicted by searching the TCDB database using BLASTP program [27, 53] with expectation value lower than 1e-05.

## Genome properties

**Fig. 3:**
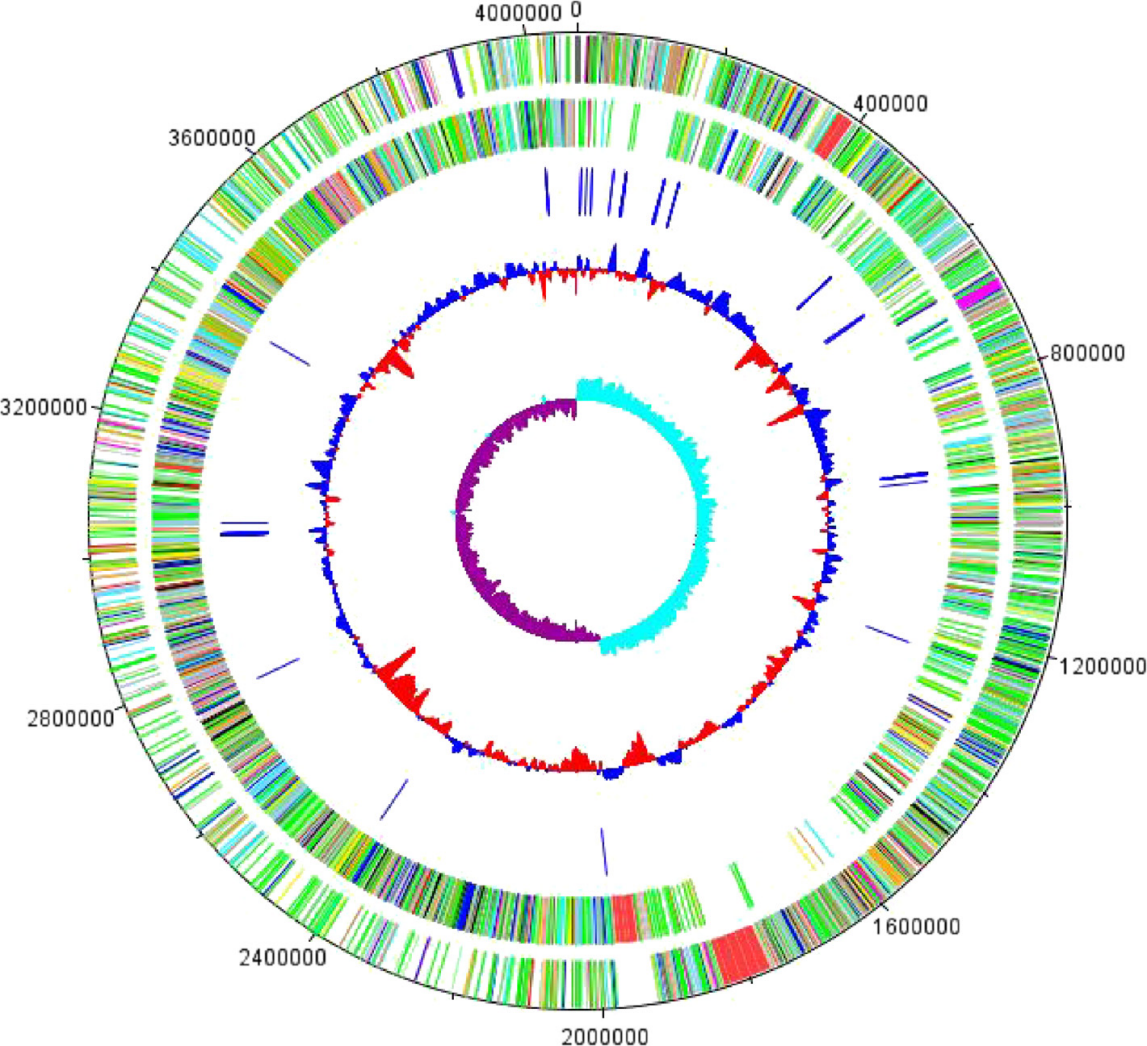
Graphic representation of circular map of the chromosome of strain LM 4–2.The map was generated with the DNAPlotter [54]. From outside to the center: the first two outer circles represent the positions of genes in the chromosome (Circle 1: plus strand, Circle 2: minus strand). Circle 3 represents tRNA genes (blue), Circle 4 represents G + C content, and Circle 5 represents GC skew

The complete strain LM 4–2 genome was composed of a circular 4,069,266 bp chromosome with an overall 43.83 % G + C content. Four thousand one hundred forty-nine ORFs, 10 sets of rRNA operons, and 84 tRNAs were predicted in the LM 4–2 genome (Table 3 and Fig. 3). Two thousand seven hundred forty-two of total 4149 predicted ORFs could be functional assignment, 1415 were annotated as hypothetical proteins. When analyzed for biological roles according to COG categories, amino acid transport and metabolism proteins accounted for the largest percent (7.18 %) of all functionally assigned proteins, followed by carbohydrate transport and metabolism proteins (6.89 %), and Transcription proteins (6.43 %). There are 687 transporter-coding and 116 redox protein-coding genes were identified in the LM 4–2 genome. The distribution of genes into COGs functional categories is presented in Table 4.

**Table 3:** Genome statistics

Attribute	Value	% of Total
Genome size (bp)	4,069,266	100.00
DNA coding (bp)	3,596,010	88.37
DNA G + C (bp)	1,811,637	44.52
Total genes	4265	100.00
Protein coding genes	4149	97.28
RNA genes	116	2.72
rRNA operons	10	0.23
Genes with function prediction	2742	64.29
Genes assigned to COGs	3111	72.94
Genes with Pfam domains	3656	85.72
Genes with signal peptides	541	12.68
Genes with transmembrane helices	778	18.24
CRISPR repeats	0	0

**Table 4:** Number of genes associated with general COG functional categories^a^

Code	Value	% age	Description
J	149	3.59	Translation, ribosomal structure and biogenesis
A	0	0.00	RNA processing and modification
K	267	6.44	Transcription
L	114	2.75	Replication, recombination and repair
B	1	0.02	Chromatin structure and dynamics
D	36	0.87	Cell cycle control, Cell division, chromosome partitioning
V	54	1.30	Defense mechanisms
T	127	3.06	Signal transduction mechanisms
M	191	4.60	Cell wall/membrane biogenesis
N	60	1.45	Cell motility
U	25	0.60	Intracellular trafficking and secretion
O	101	2.43	Posttranslational modification, protein turnover, chaperones
C	166	4.00	Energy production and conversion
G	286	6.89	Carbohydrate transport and metabolism
E	298	7.18	Amino acid transport and metabolism
F	82	1.98	Nucleotide transport and metabolism
H	114	2.75	Coenzyme transport and metabolism
I	89	2.14	Lipid transport and metabolism
P	168	4.05	Inorganic ion transport and metabolism
Q	72	1.74	Secondary metabolites biosynthesis, transport and catabolism
R	364	8.77	General function prediction only
S	347	8.36	Function unknown
-	1039	25.04	Not in COGs

^a^The total is based on the total number of protein coding genes in the annotated genome

## Conclusions

Molybdenum pollution has been reported in water and soils all around the world [55]. Some Mo-resistance bacteria can be used to immobilize soluble molybdenum toinsoluble formsalong with reducing the toxicity. In this study we presented the complete genome sequence of *Bacillus subtilis* LM 4–2, which was isolated from a molybdenum mine in Luoyang city. Due to its strong resistance to molybdate and potential utilization in bioremediation of molybdate-polluted area, we sequence the genome and try to identify the possible molecular mechanism of molybdenum-resistance. Genomic analysis of strain LM 4–2 revealed 687 transporter-coding and 116 redox protein-coding genes were separated in the genome. Three genome islands were identified in the strain LM 4–2 genome, covering 2.71 % of the whole genome. Three gene clusters were involved in the non-ribosomal synthesis of lipopeptides, such as surfactin, fengycin, and dipeptide bacilysin. Additionally, one gene clusters for subtilosin A synthesis and one gene clusters for polyketide synthesis. No CRISPRs were identified in the strain LM 4–2 genome. The complete genome sequence of strain LM 4–2 will facilitate functional genomics to elucidate the molecular mechanisms that underlie molybdenum-resistance and it may facilitate the bioremediation of molybdenum-contaminated areas.

## Additional file

Additional file 1: Table S1. The results of ANI, AAI and GGDH value between genomes of strain LM 4-2 and other 30 complete sequenced *B. subtilis* species. (DOC 58 kb)
