# Supplementary material for: Complete genome sequence of the molybdenum-resistant bacterium Bacillus subtilis strain LM 4–2
Source: Stand Genomic Sci. 2015 Dec 10;10:127. doi: 10.1186/s40793-015-0118-6 (PMC4674931; doi:10.1186/s40793-015-0118-6)
Supplement: Additional file 1: Table S1. — The results of ANI, AAI and GGDH value between genomes of strain LM 4-2 and other 30 complete sequenced B. subtilis species. (DOC 58 kb) [file 40793_2015_118_MOESM1_ESM.doc]

# Additional file 1

**Table S1.** The results of ANI, AAI and GGDH value between genomes of strain LM 4-2

and other 30 complete sequenced [*B. subtilis*](http://dx.doi.org/10.1601/nm.10618) species.

| **Species (Accession number)** | **ANI** | **AAI** | **GGDH** |
| --- | --- | --- | --- |
| [*B. subtilis*](http://dx.doi.org/10.1601/nm.10618) BEST7003 (NZ_AP012496.1) | 98.91% | 98.89% | 91.30% ± 1.96 |
| [*B. subtilis*](http://dx.doi.org/10.1601/nm.10618) BEST7613 (NZ_AP012495.1) | 98.83% | 98.41% | 90.90% ± 2.01 |
| [*B. subtilis*](http://dx.doi.org/10.1601/nm.10618) BSn5 ([NC_014976](http://www.ncbi.nlm.nih.gov/nuccore/NC_014976).1) | 98.66% | 98.94% | 89.40% ± 2.18 |
| [*B. subtilis*](http://dx.doi.org/10.1601/nm.10618) PY79 ([NC_022898](http://www.ncbi.nlm.nih.gov/nuccore/NC_022898).1) | 98.66% | 99.07% | 91.70% ± 1.91 |
| [*B. subtilis*](http://dx.doi.org/10.1601/nm.10618) QB928 ([NC_018520](http://www.ncbi.nlm.nih.gov/nuccore/NC_018520).1) | 98.96% | 99.06% | 91.80% ± 1.89 |
| [*B. subtilis*](http://dx.doi.org/10.1601/nm.10618) XF-1 ([NC_020244](http://www.ncbi.nlm.nih.gov/nuccore/NC_020244).1) | 98.20% | 98.25% | 84.90% ± 2.55 |
| [*B. subtilis*](http://dx.doi.org/10.1601/nm.10618) PS832 (NZ_CP010053.1) | 98.80% | 98.02% | 91.50% ± 1.94 |
| [*B. subtilis*](http://dx.doi.org/10.1601/nm.10618) SG6 (NZ_CP009796.1) | 98.89% | 98.21% | 90.90% ± 2.00 |
| [*B. subtilis*](http://dx.doi.org/10.1601/nm.10618) [ATCC 13952](http://doi.org/10.1601/strainfinder?urlappend=%3Fid%3DATCC+13952) (NZ_CP009748.1) | 80.19% | 80.02% | 20.80% ± 2.33 |
| [*B. subtilis*](http://dx.doi.org/10.1601/nm.10618) [ATCC 19217](http://doi.org/10.1601/strainfinder?urlappend=%3Fid%3DATCC+19217) (NZ_CP009749.1) | 80.16% | 80.06% | 20.80% ± 2.33 |
| [*B. subtilis*](http://dx.doi.org/10.1601/nm.10618) Bs-916 (NZ_CP009611.1) | 80.14% | 80.00% | 20.80% ± 2.33 |
| [*B. subtilis*](http://dx.doi.org/10.1601/nm.10618) BS49Ch (NZ_LN649259.1) | 98.99% | 98.02% | 92.10% ± 1.86 |
| [*B. subtilis*](http://dx.doi.org/10.1601/nm.10618) T30 (NZ_CP011051.1) | 92.08% | 94.72% | 49.60% ± 2.63 |
| [*B. subtilis*](http://dx.doi.org/10.1601/nm.10618) 1028 (NZ_CP011115.1) | 99.00% | 97.20% | 92.10% ± 1.86 |
| [*B. subtilis*](http://dx.doi.org/10.1601/nm.10618) HJ5 (NZ_CP007173.1) | 98.26% | 97.81% | 85.40% ± 2.52 |
| [*B. subtilis*](http://dx.doi.org/10.1601/nm.10618) UD1022 (NZ_CP011534.1) | 98.25% | 98.49% | 85.50% ± 2.51 |
| [*B. subtilis*](http://dx.doi.org/10.1601/nm.10618) TO-A JPC (NZ_CP011882.1) | 99.00% | 99.13% | 92.20% ± 1.85 |
| [*B. subtilis*](http://dx.doi.org/10.1601/nm.10618) *natto* BEST195 ([NC_017196](http://www.ncbi.nlm.nih.gov/nuccore/NC_017196).2) | 98.62% | 98.40% | 87.90% ± 2.32 |
| [*B. subtilis spizizenii*](http://dx.doi.org/10.1601/nm.4859) W23 ([NC_014479](http://www.ncbi.nlm.nih.gov/nuccore/NC_014479).1) | 92.12% | 94.72% | 49.70% ± 2.63 |
| [*B. subtilis spizizenii*](http://dx.doi.org/10.1601/nm.4859) [NRS 231](http://doi.org/10.1601/strainfinder?urlappend=%3Fid%3DNRS+231) (NZ_CP010434.1) | 92.12% | 94.77% | 49.70% ± 2.63 |
| [*B. subtilis spizizenii*](http://dx.doi.org/10.1601/nm.4859) TU-B-10 ([NC_016047](http://www.ncbi.nlm.nih.gov/nuccore/NC_016047).1) | 92.39% | 94.89% | 87.60% ± 3.16 |
| [*B. subtilis subtilis*](http://dx.doi.org/10.1601/nm.4858) 6051-HGW ([NC_020507](http://www.ncbi.nlm.nih.gov/nuccore/NC_020507).1) | 98.99% | 99.09% | 92.10% ± 1.86 |
| [*B. subtilis subtilis*](http://dx.doi.org/10.1601/nm.4858) 168 (NZ_CP010052.1) | 99.00% | 99.09% | 92.10% ± 1.86 |
| [*B. subtilis subtilis*](http://dx.doi.org/10.1601/nm.4858) AG1839 (NZ_CP008698.1) | 98.98% | 99.06% | 92.00% ± 1.87 |
| [*B. subtilis subtilis*](http://dx.doi.org/10.1601/nm.4858) [BAB-1](http://doi.org/10.1601/strainfinder?urlappend=%3Fid%3DBAB-1) ([NC_020832](http://www.ncbi.nlm.nih.gov/nuccore/NC_020832).1) | 98.25% | 98.40% | 85.20% ± 2.53 |
| [*B. subtilis subtilis*](http://dx.doi.org/10.1601/nm.4858) BSP1 ([NC_019896](http://www.ncbi.nlm.nih.gov/nuccore/NC_019896).1) | 98.76% | 98.69% | 89.60% ± 2.16 |
| [*B. subtilis subtilis*](http://dx.doi.org/10.1601/nm.4858) AG174 (NZ_CP007800.1) | 98.98% | 99.07% | 92.00% ± 1.87 |
| [*B. subtilis subtilis*](http://dx.doi.org/10.1601/nm.4858) OH 131.1 (NZ_CP007409.1) | 98.73% | 98.79% | 89.40% ± 2.17 |
| [*B. subtilis subtilis*](http://dx.doi.org/10.1601/nm.4858) RO-NN-1 ([NC_017195](http://www.ncbi.nlm.nih.gov/nuccore/NC_017195).1) | 97.95% | 98.33% | 83.30% ± 2.64 |
| [*B. subtilis subtilis*](http://dx.doi.org/10.1601/nm.4858) 3NA (NZ_CP010314.1) | 99.00% | 99.06% | 92.00% ± 1.87 |
